# Mitochondrial Dysfunction and Endoplasmic Reticulum Stress in Chronic Pain

**DOI:** 10.3390/brainsci16080896

**Published:** 2026-08-21

**Authors:** Arun Yadawa, Sufang Liu, Feng Tao

**Affiliations:** Department of Biomedical Sciences, Texas A&M University College of Dentistry, 3302 Gaston Ave., Dallas, TX 75246, USA

**Keywords:** chronic pain, mitochondrial dysfunction, endoplasmic reticulum stress, nociceptive sensitization, neuroinflammation, oxidative stress

## Abstract

**Highlights:**

**What are the main findings?**

- Defective mitochondrial function acts as a foundational trigger in the pathogenesis of chronic pain.
- ER stress drives the pathogenesis of chronic pain by altering neural circuit architecture and triggering the reactivity of both astrocytes and microglia. These cellular interactions underscore the critical role of ER-mediated neuroinflammation in sustaining persistent pain states.

**What are the implications of the main findings?**

- Integrating mitochondrial dysfunction and ER stress into a unified pathological axis may provide a useful framework for identifying shared molecular drivers of chronic pain and developing more precise therapeutic strategies.
- Deciphering the ER-mitochondrial crosstalk will open a new avenue for targeted therapeutic interventions aimed at reversing organelle-level distress in chronic pain.

**Abstract:**

Chronic pain is a major global health burden and often remains difficult to treat with current therapies, which frequently provide incomplete relief and may cause systemic side effects. As essential organelles in eukaryotic cells, mitochondria facilitate ATP synthesis and serve as key regulators of calcium homeostasis and apoptosis. Evidence points to mitochondrial dysfunction not merely as a result of trauma, but as a fundamental factor in why pain becomes persistent. On the other hand, the endoplasmic reticulum (ER) is more than just a structural component of the cell; it is a multi-functional organelle responsible for protein quality control, including folding and degradation, as well as lipid production and calcium signaling. ER dysfunction is a primary driver of various pathologies, such as cardiovascular disease, cancer, and neurodegenerative disorders. The organelle’s ability to execute its vital functions is strictly dependent on sufficient levels of ATP. Because mitochondrial and ER functions are closely interconnected through calcium exchange, ATP-dependent protein homeostasis, oxidative stress, and mitochondria-associated ER membranes, their dysfunction may act together to amplify nociceptive sensitization and pain chronification. In this review, we summarize current evidence linking mitochondrial dysfunction, ER stress, and ER-mitochondrial crosstalk to the pathogenesis of chronic pain and discuss their potentials as therapeutic targets.

## 1. Introduction

Pain is characterized as a distressing sensory and emotional event linked to, or mimicking the effects of real or possible tissue injury. While acute pain acts as a vital warning signal for bodily harm, chronic pain persists long after its initial protective function has ended or may emerge without a clear cause. Generally defined as lasting more than 3 months, chronic pain is a recurring or continuous condition that extends past the standard recovery period [1]. The occurrence of chronic pain is more frequent among women, older children, and adolescents, as well as individuals facing socioeconomic disadvantages [2]. Chronic pain typically presents as a widespread and diffuse condition, frequently occurring alongside various somatic and psychological symptoms. These comorbidities often include persistent fatigue, sleep disturbances, cognitive challenges—such as issues with memory or focus—and a depressed mood and also affects 25–35% of adults worldwide [3].

Central sensitization is a fundamental mechanism driving most chronic pain conditions, characterized by measurable functional adaptations within the nervous system [4]. At its core, this process represents a transition where the primary driver of pain shifts from peripheral tissue damage to pathological changes within the central nervous system [5]. The transition to a chronic pain state is often mediated by local and systemic inflammatory mechanisms. Evidence indicates that an altered cytokine profile enhances the sensitivity of peripheral nociceptors, effectively lowering their activation threshold and facilitating persistent pain signaling [3,5,6]. It is hypothesized that cortical disorganization reshapes the sensorimotor homunculus, leading to a more widespread and generalized experience of pain [7]. Furthermore, individuals with chronic pain often develop maladaptive ‘pain memories’. In this state, movements associated with fear or anticipation of injury can trigger a pain response even in the absence of actual nociceptive input, which can significantly exacerbate or prolong the chronic pain cycle [8].

Mitochondria are essential organelles that regulate ATP production, calcium homeostasis, redox signaling, apoptosis, and neuronal survival [9,10]. Mitochondria are pivotal in the pathogenesis and therapeutic targeting of oral inflammatory diseases, underscoring their significant role within cellular mechanisms [11]. Mitochondrial involvement not only elucidates the molecular underpinnings of disease progression but also opens avenues for novel treatment strategies [12].

Mitochondrial integrity is often compromised, and structural abnormalities are common in conditions involving oral inflammation [13], such as periodontitis, pulpitis, oral cancer, osteoradionecrosis, and Sjogren’s syndrome [14]. The mitochondrial electron transport chain (mETC) is a series of five molecular complexes through which electrons are transported and ATP is generated. Studies suggest that deficiencies within each mETC complex may contribute to the development and maintenance of chronic pain. The inhibitors of all five complexes have attenuated TNF-α and PKCε-induced inflammatory pain behaviors [15,16]. Accumulating evidence suggests that mitochondrial dysfunction is a primary driver of cellular stress and a key factor in the etiology of inflammatory and chronic pain following peripheral nerve injury [15,17,18]. These findings support mitochondrial dysfunction as an important mechanism in the development and maintenance of chronic pain.

The endoplasmic reticulum (ER) is a multifunctional organelle involved in protein folding and degradation, lipid synthesis, and calcium storage [19]. The ER membrane’s continuity with the outer nuclear membrane creates a structural link between the two, supported by shared proteins that regulate both ER shape and nuclear pore complex formation [20]. These contact sites are vital for coordinating pathways and preserving cellular integrity. While vesicles and signaling handle long-range communication, membrane contact sites provide a more direct interface that deepens the integration of cellular compartments. A prominent and well-characterized example of inter-organellar communication is found at mitochondria-associated ER membranes (MAMs). These represent a specialized subdomain of the smooth ER specifically dedicated to mitochondrial interaction. Consequently, MAMs are involved in a diverse array of physiological processes, ranging from Ca^2+^ signaling and lipid metabolism to the regulation of mitochondrial fission and inflammasome assembly [21,22,23].

The rough ER serves as the cell’s primary site for the synthesis and modification of secretory and membrane-bound proteins, overseeing processes from initial folding to glycosylation and degradation. Meanwhile, the smooth ER functions as a hub for lipid metabolism and Ca^2+^ homeostasis. The critical nature of these diverse duties ensures that any significant ER impairment translates into severe cellular injury or cell death. ER malfunction can be catastrophic, potentially leading to cell death. When protein secretion or degradation fails, un- or misfolded proteins accumulate within the lumen. These molecular defects are the hallmark of ER storage diseases [24]. ER proteostasis is maintained by a stringent quality control filter that exports functional proteins to the Golgi while degrading those that are misfolded. Stress-induced protein aggregation can bypass this filter, initiating the unfolded protein response (UPR)—a collection of signaling pathways designed to mitigate cellular damage. If this adaptive response fails to resolve the underlying stress, the UPR switches from a cytoprotective to a pro-apoptotic role, leading to irreversible cell death [25,26]. These energy-intensive operations are powered by ATP, which is essential not only as a universal fuel but specifically for the extensive protein phosphorylation that governs ER signaling and proteostasis.

Consequently, a more profound understanding of the precise molecular mechanisms underlying mitochondrial dysfunction and ER stress in chronic pain is essential. Elucidating these pathways will be pivotal in developing novel, highly effective analgesic interventions. Although mitochondrial dysfunction and ER stress have been implicated in chronic pain, they are frequently examined as separate mechanisms. Important questions remain regarding whether these organelle responses initiate or maintain chronic pain, how their effects vary across cell types and disease stages, and how ER and mitochondrial communication contributes to neuronal sensitization and neuroinflammation. Moreover, direct evidence from human chronic pain remains limited. Therefore, this review aims to integrate current evidence on mitochondrial dysfunction, ER stress, and their crosstalk, with particular emphasis on mitochondria-associated ER membranes, and to evaluate how these interconnected pathways contribute to pain chronification and may inform future therapeutic strategies.

The present review distinguishes itself by integrating mitochondrial dysfunction, ER stress, and ER-mitochondrial crosstalk into a unified organelle-stress framework across chronic pain conditions. It further links these mechanisms to therapeutic strategies while addressing cell-type specificity, disease stage, delivery limitations, and the scarcity of human evidence. Rather than considering mitochondrial dysfunction and ER stress as separate processes, this review emphasizes how their reciprocal interactions may drive pain persistence and chronification.

### Literature Search and Selection

A narrative literature search was conducted using PubMed, Web of Science, and Google Scholar through 2000 and 2026 year. Search terms included combinations of “chronic pain,” “neuropathic pain,” “inflammatory pain,” “mitochondrial dysfunction,” “mitochondrial bioenergetics,” “mitophagy,” “endoplasmic reticulum stress,” “unfolded protein response,” “ER–mitochondrial crosstalk,” and “mitochondria-associated ER membranes.” Relevant original research articles and reviews published in English were identified, with emphasis on mechanistic studies directly examining mitochondrial dysfunction, ER stress, or their interaction in chronic pain. Because this was a narrative review, meta-analyses were not performed.

## 2. Mitochondrial Dysfunction in Chronic Pain

Mitochondria serve as the cell’s principal energy transducers, accounting for over 90% of total ATP production. Due to the exceptional metabolic requirements of neural tissue, mitochondrial homeostasis is a critical determinant of neuronal health and nervous system function [27]. Given their central role in regulating calcium buffering, redox signaling, and apoptotic pathways, mitochondria represent a critical vulnerability in the nervous system. Disruption of these multifaceted mitochondrial interactions is a potent driver of neuronal dysfunction [28]. The pivotal role of mitochondria in maintaining neuronal integrity is underscored by the fact that their dysfunction is a recognized keystone of many neurological pathologies, most notably within the landscape of chronic neurodegeneration [28,29].

Emerging evidence identifies mitochondrial dysfunction as a primary driver in the pathogenesis of chronic pain, where bioenergetic failure contributes to both the initial development and the subsequent maintenance of central sensitization [27,28,30,31]. Mitochondrial impairment serves as a multi-modal driver of chronic pain; beyond directly destabilizing neuronal membrane potentials and viability, it exacerbates the maintenance of pain signaling by recruiting neuroinflammatory pathways and activating resident immune cells [32]. Mitochondrial impairment is increasingly recognized as a systemic driver of chronic pain, where peripheral metabolic deficits provide a pathological framework that facilitates the transition from acute to persistent hypersensitivity [33,34].

Mitochondria represent a multifaceted organelle network indispensable for cellular survival, governing critical homeostatic functions such as energy transduction, the regulation of mitochondrial reactive oxygen species (ROS), and the maintenance of calcium homeostasis [35]. The physiological function of mitochondria is maintained through a delicate dynamic equilibrium, governed by a sophisticated hierarchical quality control system that preserves mitochondrial health and network stability [36]. Impairment at any stage of mitochondrial quality control results in functional decline, typically manifested by mitochondrial DNA (mtDNA) leakage, morphological fragmentation, and a loss of mitochondrial membrane potential (MMP) [27,37]. These collective defects represent a unified pathological state that serves as a central driver in various conditions, most notably chronic pain.

Mitochondrial oxidative capacity and antioxidant responses display significant sexual dimorphism. Physiologically, females maintain higher baseline MnSOD and GSH antioxidant enzyme activity compared to males [38]. Sex-dependent cell specificity critically shapes mitochondrial involvement in pain pathogenesis. While male neuropathic pain is predominantly mediated by the microglial mtROS–NLRP3 signaling axis [35,39], female pain phenotypes often rely on T cells and astrocytes—such as Drp1-dependent astrocytic mitochondrial fission in female bone cancer pain [38,40,41]. Nevertheless, this dichotomy is complex, with female DRG neurons demonstrating heightened bioenergetic vulnerability and mtROS buildup in specific neuropathic contexts [41,42]. Ultimately, these distinct biological profiles heavily influence the outcome of mitochondria-targeted therapies.

### 2.1. The Involvement of Mitochondrial Dysfunction in Chronic Pain

Manifesting in diverse clinical forms, chronic pain is driven by various complex mechanisms. Mitochondria are vital for preserving neuronal homeostasis, as they regulate essential metabolic processes and generate energy in the form of ATP through oxidative phosphorylation (OxPhos). Beyond energy production, mitochondria are fundamental regulators of various cellular activities, including the maintenance of calcium homeostasis, the modulation of ion channel dynamics, and the orchestration of reactive oxygen species signaling [43]. Disruptions in these mitochondrial functions are closely associated with the development of chronic pain. In clinical settings, nearly 70% of individuals diagnosed with hereditary mitochondrial disorders report experiencing chronic pain [44]. Emerging research highlights mitochondrial dysfunction as a central player in this landscape, demonstrating how mitochondrial failure can trigger or reinforce the persistence of pain signals by disrupting cellular homeostasis at several stages. Under physiological conditions, mitochondria release O^2−·^; hypoxic conditions exaggerate release of O^2−·^ because of mitochondrial inhibition. Mitochondria typically transfer electrons via the respiratory chain to generate water along with ATP production. This electron transport chain is hampered by oxygen deprivation resulting in electron leakage. This electron leakage produces O^2−·^ leading to the oxidative decarboxylation of α-ketoglutarate and the subsequent inhibition of its cofactor, prolyl hydroxylase domain-2 (PHD2). PHD2 facilitates the proteasomal degradation of hypoxia-inducible factor 1α (HIF-1α) by its hydroxylation [45].

Previous studies have demonstrated the involvement of mitochondrial dysfunction and the production of mitochondrial ROS as key factors in the development of neurodegenerative disorders like Alzheimer’s disease and Parkinson’s disease [46,47,48]. Emerging evidence suggests a strong correlation between mitochondrial dysfunction/hyperfunction and diverse pain conditions, including inflammatory pain and neuropathic pain [27,28]. In recent years the role of mitochondria in pain is elucidated in many pain models. TG derived mitochondria transplantation in neurons significantly alleviated the inflammation-induced orofacial pain [49]. Substantial research into peripheral nerve injury models has established mitochondrial dysfunction as a pivotal player in maintaining chronic pain. Following peripheral nerve damage, mitochondrial defects manifest within critical nodes of the pain pathway, such as the spinal cord dorsal horn, suggesting that cellular energy failure is linked to central sensitization [42]. As metabolic centers, mitochondria regulate neuronal function at multiple levels. Within primary afferents, uninterrupted ATP production is critical for pain maintenance. Specifically, disrupting the mETC at any of its five complexes induces ATP-dependent analgesia in HIV-associated pain models, highlighting the energetic requirements of chronic nociception [15]. Clinical data from trigeminal neuralgia patients reveal compensatory hyperactivity in mitochondrial respiratory chain function, as measured in serum samples [50]. Conversely, recent research using trigeminal neuralgia models identified a significant decline in the NAD^+^/NADH ratio within the trigeminal ganglion. This metabolic imbalance was successfully corrected by supplementing with the NAD^+^ precursor nicotinamide riboside, which restored mitochondrial efficiency and mitigated pain by activating the NAD^+^-dependent deacetylase SIRT1 [51].

Numerous preclinical investigations have established a connection between mitochondrial dysfunction—specifically diminished ATP synthesis—within sensory neurons and the persistence of chronic pain in rodent models, particularly those involving chemotherapy-induced neuropathy [52,53]. Furthermore, the mitochondrial protein ATPSc-KMT has been identified as a key regulator in this process. As a lysine-specific methyltransferase, it methylates the c-subunit of ATP synthase, thereby modulating OxPhos activity and facilitating the transition to chronic pain [54]. Interestingly, sensory neuron OxPhos undergoes specific adaptations during temporary inflammatory episodes; the successful resolution of this pain appears to rely on the active transfer of mitochondria from macrophages to sensory neurons [55]. Mitochondria serve as the primary source of ROS, which are key contributors to both the initial development and the long-term maintenance of neuropathic pain [56].

A recent study reported that in a rat model of TMJ-OA pain at 4 weeks, the key genes involved in ROS production (Nox1, Nox4, and Cyp1a1), mitochondrial biogenesis and energy conversion (Ppargc1a/Pgc-1α, Slc25a4/Ant1), and energy metabolism (Slc2a4/Glut4, Cpt1b, Pfkm) were significantly upregulated. These upregulations correlated with enhanced biological processes related to energy and ATP production, calcium flux, oxidative stress, and response to mechanical stimuli. At later time point of TMJ-OA at 12 weeks, a notable decline in energy-related biological processes was observed, specifically affecting cellular respiration, the assembly of oxidative respiratory chain complexes, and overall electron transport chain functionality. Downregulation of pathways includes OxPhos, the TCA cycle, glycolysis/gluconeogenesis, fatty acid degradation, and mitophagy. Study also revealed decline in the expression of genes related to mitophagy, mitochondrial membrane transport, mitochondrial dynamics, and the respiratory chain complex [57]. These findings suggest that the abnormal mechanical stress imposed on the TMJ may impair chondrocyte mitochondrial function and thereby affects cellular energy metabolism. Mitochondrial function was compromised in the brainstem as well as in the cortex of trigeminal inflammatory compression orofacial pain mouse model [58].

The mitochondrial dysfunction has also been implicated in the mouse model of inflammatory pain. The mitochondrial dysfunction in trigeminal ganglia (TG) of inflammatory pain mice was evidenced by decreased expression of genes related to mitochondrial metabolism, reduced mitochondrial membrane potential, and decreased oxygen consumption rate in TG neurons. Impaired mitophagy in TG neurons was also observed in inflammatory conditions [49]. Impaired autophagy in the spinal cord sustains neuropathic pain [59]. Some recent studies suggest mitochondrial subpopulations-one specialized for energy production and other for molecular biosynthesis [60,61,62].

Peripheral nerve injury suppresses the spinal PGC-1α/NRF1/TFAM signaling axis, leading to diminished mtDNA copy numbers and ATP depletion [35,63]. Accompanied by defective mitophagy, elevated mtROS levels activate the NLRP3 inflammasome within spinal dorsal horn glia, creating a self-reinforcing loop of mitochondrial damage and mtROS generation that promotes microglial M1 polarization [35]. Targeting these pathways by enhancing mitochondrial quality control and blocking downstream inflammatory cascades offers a potent analgesic approach [63,64]. Mitochondrial dysfunction within primary sensory neurons serves as a common pathogenic driver of chemotherapy-induced peripheral neuropathy (CIPN), uniting the varied primary mechanisms of different chemotherapeutic agents [65,66]. Chemotherapy severely disrupts mitochondrial homeostasis. Specifically, oxaliplatin and vincristine induce excessive mitochondrial fission by promoting Drp1 Ser616 phosphorylation while downregulating Mfn1 and Mfn2 [30,67]. The resulting network fragmentation initiates a vicious cycle: fission drives mtROS generation, which subsequently feeds back to enhance Drp1 activity [30]. In patients with fibromyalgia, systemic bioenergetic deficits are consistently observed across multiple tissue types, including peripheral blood mononuclear cells, skin biopsies, muscle biopsies, and dermal fibroblasts. Marked by diminished ATP production, CoQ10 deficiency, and heightened oxidative stress, this mitochondrial impairment correlates directly with disease severity and widespread pain scores [68,69].

### 2.2. Mechanisms Underlying Mitochondrial Dysfunction in Chronic Pain (Figure 1)

#### 2.2.1. Mitochondrial Bioenergetic Dysfunction

Mitochondria maintain bioenergetic stability in high-energy-demand neurons by performing efficient OxPhos. This process, driven by the mETC on the inner mitochondrial membrane, provides over 90% of the cellular ATP necessary for physiological function [70,71]. The ATP generated by mitochondria is vital for the physiological integrity of the nervous system, providing the energy required to maintain resting potentials, re-establish ionic equilibrium post-excitation, and facilitate the complex molecular changes underlying synaptic plasticity [15,72]. The transition to a bioenergetic crisis is often rooted in the impairment of mETC complexes I and IV, which undermines the efficiency of OxPhos [15]. This metabolic collapse suppresses the ATP-dependent Na^+^/K^+^-ATPase, triggering a rise in intracellular Na^+^ and Ca^2+^ concentrations—a hallmark of the membrane depolarization seen in chronic pain states [42]. These changes elevate spontaneous neuronal firing and hyperexcitability—hallmarks of neuropathic pain. Such dysfunction often manifests as hyperactivity; for example, the upregulation of the mitochondrial methyltransferase ATPSc-KMT (FAM173B) in sensory neurons enhances OxPhos, thereby promoting chronic pain [73]. Consequently, both bioenergetic failure and maladaptive metabolic surges can destabilize cellular energy homeostasis, triggering the transition into a chronic pathological state.

**Figure 1:**
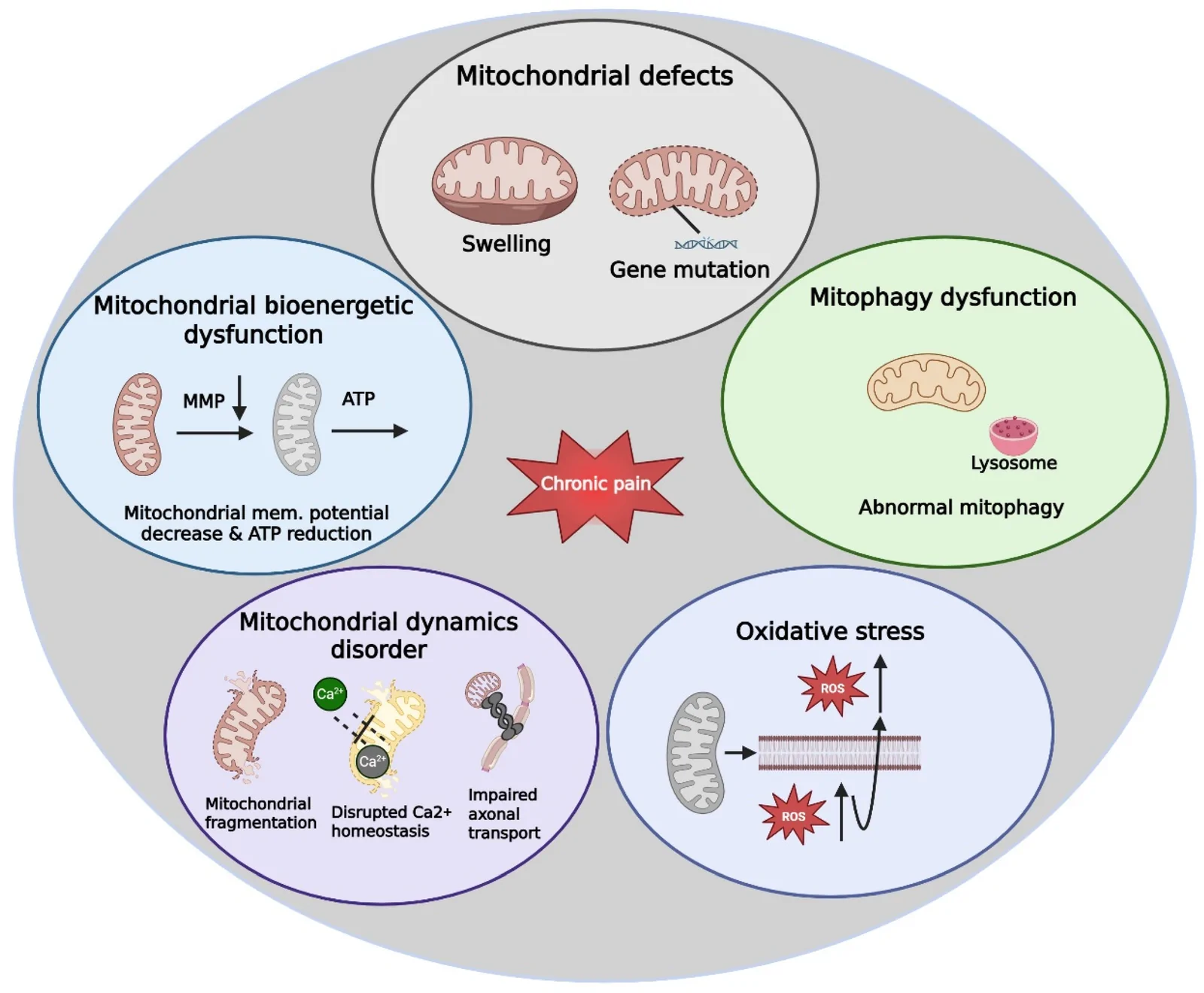
Mitochondrial dysfunction mechanisms in chronic pain. Mitochondrial defects: Mitochondrial swelling contributes to mitochondrial dysfunction via the opening of mitochondrial permeability transition pore; mitochondrial gene mutation leads to energy deficit in neurons and thereby apoptosis. Mitophagy dysfunction: Impaired mitophagy results in the accumulation of damaged mitochondria which again leads to ATP deficiency in the neurons. Mitochondrial bioenergetic dysfunction: Bioenergetic impairment is associated with reduced mitochondrial membrane potential and decreased ATP production. Mitochondrial dynamics disorder: The imbalance between mitochondrial fission and fusion of the mitochondria promotes mitochondrial fragmentation, disrupts Ca^2+^ homeostasis and impairs mitochondrial transport within neurons. Oxidative stress: Under pathological conditions, excessive ROS production induces oxidative stress, thereby compromising neuronal function and excitability.

#### 2.2.2. Oxidative Stress

Energy failure is frequently accompanied by excessive mitochondrial reactive oxygen species (mtROS) production and subsequent oxidative stress. While mitochondria naturally generate small, regulated amounts of mtROS as a byproduct of OxPhos, pathological states exacerbate electron leakage. Specifically, a small percentage of electrons escape the mETC—primarily at Complexes I and III—to form superoxide (O_2_^.−^), which is subsequently converted into other reactive species [42,59]. Under physiological conditions, mtROS acts as a vital second messenger, regulating essential processes such as neuronal differentiation, synaptic pruning, and neurotransmitter release [30,42]. However, a direct and immediate consequence of a faulty mETC is the pathological overproduction of mtROS. Dysfunctional mETC components facilitate electron leakage, which reacts with molecular oxygen to generate massive quantities of reactive species [30]. Disruption of this redox balance culminates in severe oxidative stress, where excessive mtROS inflict oxidative damage upon lipids, proteins, and nucleic acids [30,42]. Crucially, this damage extends to mtDNA itself, precipitating a ‘vicious cycle’ of ROS-induced ROS release that further compromises mitochondrial integrity and exacerbates cellular injury [42]. Beyond the direct degradation of mitochondria and intracellular macromolecules, oxidative stress induces sensory neuron hyperexcitability. This occurs through the targeted activation of nociceptive ion channels, most notably TRPA1, which directly facilitates the generation and transmission of pain signals [27].

#### 2.2.3. Calcium Overload and Neuroinflammation

Mitochondria also serve as critical, high-capacity buffers essential for maintaining intracellular calcium homeostasis. Under physiological conditions, mitochondria regulate cytosolic Ca^2+^ levels through a balanced process of uptake and efflux: they sequester Ca^2+^ via the mitochondrial calcium uniporter located on the inner membrane and release it back into the cytosol through the Na^+^/Ca^2+^ exchanger [27,37]. Mitochondria-associated ER membranes (MAMs) represent specialized structural domains that facilitate the efficient capture of Ca^2+^ released from the ER. During ER stress, this mechanism leads to an excessive efflux of Ca^2+^ and its subsequent influx into the mitochondrial matrix. Furthermore, pathological conditions such as sustained neuronal hyperexcitability can cause a chronic elevation of cytosolic Ca^2+^ levels. Consequently, the concentration of free Ca^2+^ within the mitochondrial matrix can surge dramatically [31]. Mitochondrial calcium overload directly inhibits mETC function and exacerbates the production of mtROS. Furthermore, it serves as a primary trigger for the opening of the mitochondrial permeability transition pore (mPTP) [30]. The activation of the mPTP leads to the rapid collapse of the mitochondrial membrane potential and the subsequent release of pro-apoptotic factors, such as cytochrome c. This sequence ultimately initiates apoptosis [27,31].

Damaged mitochondria can promote inflammatory response. Following sustained mPTP opening, various components of dysfunctional mitochondria—including mtDNA, cardiolipin, and formyl peptides—are released into the cytosol. Once liberated, these molecules act as mitochondrial damage-associated molecular patterns (mtDAMPs), triggering potent downstream inflammatory responses [42].

#### 2.2.4. Mitochondrial Dynamics and Mitophagy Dysfunction

To counteract mitochondrial decay, cells employ a homeostatic lifecycle consisting of mitophagy and biogenesis. Mitophagy acts as the selective autophagic clearance of compromised organelles, while mitochondrial biogenesis drives the production of fresh, healthy mitochondria to offset these losses. The imbalance between inadequate mitophagy and suppressed biogenesis creates a bottleneck in mitochondrial maintenance [71]. By failing to clear damaged organelles or generate functional replacements, the cell becomes trapped in a state of persistent mitochondrial dysfunction, fueling both bioenergetic failure and oxidative damage.

A dynamic equilibrium of fusion and fission governs the mitochondrial population. Mediated by Mfn1/2 and Opa1, fusion promotes the integration of the mitochondrial network [27]. This structural union serves as a metabolic adaptation, boosting the MMP and maximizing OxPhos output to meet rising energy requirements. On the other hand, Drp1-mediated fission ensures proper mitochondrial division following biogenesis [30]. This process also provides the cell with structural flexibility; during environmental shifts or cellular damage, fission allows mitochondria to be redistributed, supporting the metabolic changes necessary for cellular survival and adaptation [27]. Pathological conditions often skew mitochondrial dynamics toward fission through the upregulation of Drp1 [30,74]. Under Ca^2+^ overload, calcineurin dephosphorylates Drp1 at Ser637, effectively removing the inhibitory brake on fission [75]. This leads to extensive mitochondrial fragmentation, leaving the cell with small, depolarized mitochondria that cannot meet metabolic energy demands. The consequence is a network of fragmented, depolarized mitochondria that lack the functional capacity to maintain adequate ATP production or energy homeostasis.

#### 2.2.5. Mitochondrial Defects

Structurally, healthy mitochondria exhibit an elliptical shape. Pathological morphological changes—including vacuolization and swelling—impair proton gradient maintenance, leading to a loss of mitochondrial membrane potential and reduced ATP synthesis [76,77]. Transmission electron microscopy reveals that mitochondrial structural alterations heavily contribute to neuropathic pain: swollen and vacuolated mitochondria accumulate in peripheral sensory axons [27] and DRG neurons during CIPNP [56], whereas spinal dorsal horn mitochondria respond to neuropathic pain stress through fragmentation and proliferation [74]. Furthermore, mtDNA mutations disrupt electron transport chain complexes, elevating ROS production and blunting ATP generation [78]. In fibromyalgia syndrome (FMS), this mtDNA-driven bioenergetic impairment directly correlates with clinical symptoms like myalgia [79].

### 2.3. Cell-Type Specific Mitochondrial Dysfunction in Chronic Pain

Although mitochondrial dysfunction represents a shared hallmark of chronic pain, distinct functional deficits emerge according to cell type—whether neuronal, glial, or supporting.

Neuronal mitochondrial pathology centers on a bioenergetic crisis that promotes hyperexcitability. Unable to meet high metabolic demands, neurons experience rapid ATP depletion [15,27,70] coupled with disrupted calcium dynamics—driven by mitochondrial calcium uniporter upregulation and Na^+^/Ca^2+^ exchanger (NCLX) downregulation—that lowers the threshold for ectopic discharge [37,72]. Furthermore, Drp1-mediated mitochondrial fission promotes the accumulation of fragmented mitochondria, ultimately halting normal axonal transport [29,30,74]. In contrast, glial mitochondrial impairment primarily fuels neuroinflammatory pathways. Here, mtROS functions as an active upstream signal for NLRP3 inflammasome activation, triggering the processing of IL-1β and IL-18 [35,39,80]. Furthermore, astrocytic dysfunction marked by Drp1-dependent mitochondrial fragmentation compromises both the astrocyte-neuron lactate shuttle and glutamate uptake [35,50,58,81].

Distinctly, peripheral Schwann cells exhibit a phenotype characterized by metabolic support failure. Their dysfunction involves maladaptive shifts in lipid metabolism and glycolysis, which are frequently triggered by low-density lipoprotein receptor-related protein-1 (LRP1) deficiency [82,83]. This metabolic reprogramming leads to the toxic accumulation of acylcarnitines. Consequently, Schwann cell mitochondrial dysfunction drives secondary axonal degeneration and demyelination, promoting persistent pain even without direct neuronal injury [66,82,84]. Similarly, bioenergetic deficits in peripheral tissues—including skeletal muscle, blood cells, and the gastrointestinal tract—have been implicated in nociplastic pain mechanisms [34,85].

## 3. ER Stress in Chronic Pain

Persistent pain contributing to neuroinflammatory states and protein homeostasis disturbances may lead to UPR and ER stress. The UPR represents an evolutionarily conserved signaling network triggered by ER stress, a condition arising when the accumulation of misfolded or unfolded proteins exceeds the organelle’s folding capacity [86]. ER stress signaling is coordinated by three primary membrane-bound sensors: IRE1, PERK, and ATF6, which initiate the adaptive and maladaptive phases of the UPR [87]. Under physiological conditions, the ER stress sensors IRE1, PERK, and ATF6 are maintained in an inhibited state via binding to the chaperone BiP/GRP78 [88]. The competitive recruitment of BiP to accumulating misfolded proteins triggers its dissociation from these sensors, effectively activating the UPR signaling network [89]. Activated IRE1 undergoes dimerization and autophosphorylation, initiating an endoribonuclease activity that processes XBP1 mRNA into its active spliced form (XBP1s). This transcription factor subsequently drives the expression of genes essential for ER-associated degradation (ERAD) and chaperone-mediated folding [90]. The dual nature of IRE1 signaling suggests that unresolved ER stress may be a primary driver of neuroinflammation; by facilitating the activation of JNK and NF-κB, IRE1 links neuronal proteostatic strain to the inflammatory cascades that sustain central sensitization [91]. Activation of PERK leads to the phosphorylation of eIF2α, which transiently suppresses global protein translation to alleviate the protein-folding burden on the ER lumen [92]. Despite a global decrease in protein synthesis, the PERK-eIF2α axis prioritizes the translation of ATF4. This critical transcription factor serves as a metabolic master regulator, upregulating cytoprotective genes that bolster the cell’s antioxidant defenses and amino acid availability during periods of ER strain [93]. Under persistent ER stress, ATF4 facilitates the expression of the pro-apoptotic factor CHOP, signaling a transition from cellular adaptation to programmed cell death [94]. In parallel, ATF6 undergoes Golgi-dependent proteolytic cleavage by site-1 protease and site-2 protease, releasing an active cytosolic fragment that transcriptionally upregulates ER chaperones and ERAD components to strengthen proteostatic recovery [95,96].

### 3.1. The Involvement of ER Stress in Chronic Pain

Modern perspectives on chronic pain have shifted from viewing it as a static symptom of injury to recognizing it as a state of active maladaptive plasticity, characterized by profound biochemical and cellular shifts across neural circuits [97,98]. In rodent models of neuropathic pain, such as the spinal nerve ligation (SNL) model, there is substantial evidence that ER stress and the subsequent activation of the unfolded protein response (UPR) occur within the spinal cord. A study by Zhang et al. demonstrated that SNL-induced nerve injury leads to a robust upregulation of the BiP (GRP78) and the active, spliced form of XBP1 specifically in the neurons of the spinal dorsal horn of rat [99]. The induction of these UPR markers signifies a state of unresolved ER stress, likely driven by the aberrant accumulation of misfolded proteins in neurons following nerve trauma. Pharmacological intervention with the chemical chaperone tauroursodeoxycholic acid (TUDCA) significantly reduced the expression of ER stress markers and attenuated mechanical allodynia in SNL rats, highlighting the role of proteostatic strain in pain maintenance [100,101]. Notably, the intrathecal delivery of thapsigargin to healthy rats—which triggers ER calcium depletion and subsequent protein misfolding—was sufficient to induce mechanical hyperalgesia, effectively phenocopying the behavioral profile of chronic pain through ER stress induction [102]. Chronic inflammation and the accelerated synthesis of extracellular proteins in arthritis impose a significant proteostatic burden on chondrocytes and synoviocytes, manifesting as ER stress and the production of dysfunctional matrix proteins [103].

ER stress arises from the accumulation of misfolded or unfolded proteins, which compromises the organelle’s protein-folding capacity and disrupts cellular proteostasis. Although the three major ER stress pathways, including PERK/eIF2α, IRE1α/XBP1, and ATF6, are initially activated to restore cellular homeostasis, chronic activation can shift the UPR from a protective response to a driver of pathology. ER stress is increasingly documented as a common pathological factor in various pain states, including cancer-induced bone pain [104], central post-stroke pain [105], and diabetic peripheral neuropathy [106]. Its role in the chronification of pain is mediated through several key mechanisms, most notably neuroinflammation [105], neuronal sensitization [107], and maladaptive synaptic plasticity [108]. ER stress triggers nociceptive signaling via the IRE1α–XBP1s axis, which facilitates increased prostaglandin synthesis and correlates with pain-related behaviors in vivo [109]. Furthermore, this stress response causes microglial activation and the subsequent release of pro-inflammatory mediators within the spinal cord, significantly intensifying pain perception [110]. A prominent consequence of ER stress is the disturbance of calcium homeostasis. By impairing the efficacy of calcium-regulatory proteins, ER stress causes abnormal intracellular Ca^2+^ signaling, which plays a major role in neuronal dysfunction [111]. This dysregulation involves aberrant Ca^2+^-induced Ca^2+^ release, a phenomenon documented in inflammatory pain models that results in heightened cytosolic Ca^2+^ transients and subsequent hyperexcitability of nociceptive neurons [112,113,114]. Furthermore, disrupted calcium signaling reciprocally reinforces ER stress, establishing a deleterious feed-forward loop that perpetuates chronic pain states [115]. Emerging evidence suggests that ER stress within the peripheral nervous system-most notably in the DRG-functions as a critical modulator of nociceptive transmission [114,116]. ER stress in spinal dorsal horn plays a pivotal role in inflammatory and neuropathic pain [117,118,119].

A recent study indicates that partial infraorbital nerve transection—a standard model for trigeminal neuropathic pain—triggers elevated levels of ER stress in the TG and pharmacological inhibition of ER stress significantly alleviated the mechanical allodynia [120]. Chronic activation of the PERK/eIF2α pathway triggers CHOP-mediated apoptosis within dorsal horn inhibitory neurons [121], IRE1α/XBP1 pathway in microglia facilitates the secretion of TNF-α and IL-1β, thereby escalating the neuroinflammatory response [122,123], ATF6-mediated induction of GRP78 initially aims to restore proteostasis [124], yet this protective response ultimately fails under chronic pain conditions [125,126,127]. Emerging evidence suggests that neurotrophic factors provide a dynamic layer of regulation, modulating ER stress resilience [128]. Distinct neurotrophic signaling pathways exhibit pathway-specific modulation of the unfolded protein response (UPR); for instance, BDNF facilitates ATF6-mediated adaptation in Alzheimer’s models [129], while Glial Cell Line-Derived Neurotrophic Factor (GDNF) selectively mitigates PERK hyperactivation in the context of diabetic neuropathy [130]. Evidence from chronic post-surgical pain models demonstrates a significant upregulation of the UPR markers GRP78 and CHOP, reflecting a state of unresolved ER stress [116,125]. Accumulating evidence indicates that chronic pain conditions, particularly those with neuropathic components, are defined by profound proteostasis disturbances. From the activation of UPR sensors to the therapeutic efficacy of chemical chaperones, these findings suggest that chronic pain involves a molecular pathology that significantly overlaps with the mechanisms of protein misfolding diseases. The transition to chronic pain is characterized by a state of persistent cellular stress; prolonged high-frequency neuronal discharge and chronic inflammation likely exceed the protein-folding capacity of the ER, leading to the accumulation and aggregation of misfolded proteins within pain pathways [131]. Chronic neuronal hyperexcitability in pain-processing circuits drives a state of metabolic exhaustion; the resulting mitochondrial dysfunction and ROS production impair the ATP-dependent chaperone machinery, thereby facilitating the accumulation of oxidatively damaged, misfolded proteins [132,133,134]. A recent study reported the elevated ER stress markers in the serum of fibromyalgia syndrome patients [135].

Neuronal excitability and ER stress intersect at calcium. The ER is a major Ca^2+^ store, and many ER chaperones are Ca^2+^-dependent [136]. Neuronal overactivation during chronic pain central sensitization perturbs Ca^2+^ dynamics across the cytosol and ER; the resulting depletion of luminal ER Ca^2+^ impairs protein folding fidelity and initiates the UPR [137]. In the context of unresolved nerve injury, the UPR shifts from a transient adaptive response to a state of chronic maladaptation [138]. This persistent activation, mediated by the PERK/eIF2α pathway, not only reduces overall protein translation but also drives the pathological synaptic reorganization and depletion of inhibitory interneurons that characterize chronic pain [139,140]. Chronic neuroinflammation may precipitate a state of ‘inflammation-induced proteotoxicity’, where the persistent inflammatory milieu overwhelms the cellular protein quality control machinery and directly contributes to the accumulation of misfolded proteins within pain pathways [141]. Consequently, the chronic neuroimmune activation characteristic of pain states serves as a plausible driver of protein misfolding leading to ER stress.

### 3.2. Mechanisms Underlying ER Stress in Chronic Pain (Figure 2)

#### 3.2.1. ER Stress and Inflammation

Nociceptive neurons and inflammatory pathways engage in bidirectional signaling, with neurons actively shaping the inflammatory milieu [142]. Given that the ER facilitates the extensive protein synthesis required for inflammation [109], excessive biosynthetic demands often result in ER stress and UPR activation [143]. Activation of the PERK-eIF2α-ATF4 axis in cancer pain models increases spinal inflammatory markers (TNF-α, IL-1β, and IL-6), an effect that can be rescued by the selective inhibitor GSK2606414 [143]. In vitro data further implicate the NLRP3 inflammasome as a key partner to ER stress, contributing to neuropathic pain through specialized inflammatory signaling [144].

**Figure 2:**
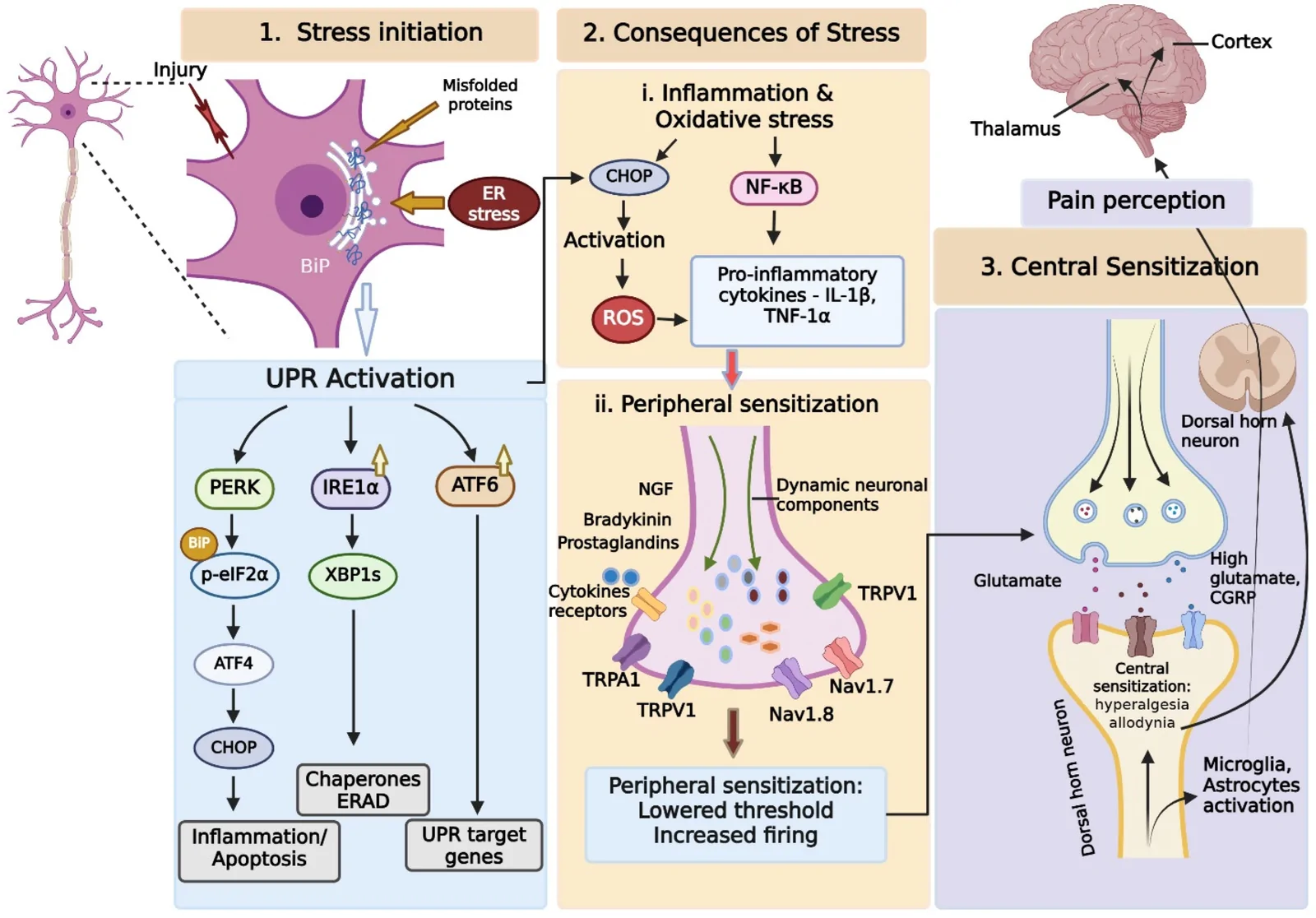
ER Stress Pathways in Nociceptive Processing and Pain Sensitization. (1) Stress Initiation and UPR Activation: Following tissue injury or peripheral nerve insult, primary sensory neurons within the Dorsal Root Ganglion (DRG) experience an accumulation of misfolded proteins, triggering ER stress. This leads to the dissociation of Binding Immunoglobulin Protein (BiP) from three major transmembrane sensors, activating the Unfolded Protein Response (UPR) pathways: (a) The PERK pathway promotes the phosphorylation of eIF2α, driving ATF4 translation and downstream CHOP induction to modulate inflammatory and apoptotic signaling. (b) The IRE1α pathway initiates the unconventional splicing of XBP1 mRNA to transcriptionally upregulate molecular chaperones. (c) The ATF6 pathway undergoes proteolytic cleavage to translocate to the nucleus and activate target survival or stress-response genes. (2) Consequences of ER Stress and Peripheral Sensitization: (A) Inflammation & Oxidative Stress: Chronic UPR activation leads to CHOP- and NF-κB-mediated expression of pro-inflammatory cytokines IL-1β, TNFα, IL-6 and increased generation of ROS. (B) Peripheral Sensitization: Accumulating inflammatory mediators (including prostaglandins, NGF, and bradykinin) bind to their respective receptors on the nociceptor membrane. This signaling cascade lowers the activation threshold and increases the membrane traffic of key ion channels, including transient receptor potential channels (TRPV1, TRPA1) and voltage-gated sodium channels Na_V_1.7, Na_V_1.8, resulting in a state of heightened peripheral firing. (3) Central Sensitization & Pain Perception: Increased nociceptive input from the periphery drives excessive release of excitatory neurotransmitters (e.g., glutamate) and neuropeptides (CGRP) into the synaptic cleft at the spinal cord dorsal horn. This persistent bombardment activates post-synaptic dorsal horn neurons and triggers surrounding microglia and astrocytes to release further pro-inflammatory cytokines IL-1β, TNFα. Together, these alterations establish central sensitization, a state of pathological hyperexcitability clinically manifesting as hyperalgesia and allodynia. Ascending pathways project these amplified pain signals via the thalamus to the cerebral cortex, leading to the conscious perception of chronic pain.

#### 3.2.2. ER Stress and Autophagy

Autophagy and ER stress are reciprocally linked mechanisms that govern protein homeostasis in the nervous system. By regulating the balance between protein synthesis and degradation, autophagy serves as a key mediator of cell survival [145]. Efficient autophagy mitigates ER stress by removing misfolded proteins, whereas its inhibition can trigger ER-stress-mediated cell death [146]. Autophagy is both a protective factor and, when impaired, a contributor to chronic pain [147]. Research using the spinal nerve ligation model shows that ER stress triggers an increase in spinal LC3 and p62 expression, highlighting the interplay between organelle stress and autophagic signaling in the progression of neuropathic pain [117]. Conversely, pharmacological inhibition of ER stress effectively lowers the levels of autophagy markers [148]. Autophagy-driven astrocyte activation serves as a critical mechanism underlying the hypersensitivity and pain seen in diverse clinical conditions [142,149].

#### 3.2.3. ER Stress and Oxidative Stress

Oxidative stress is a key mediator in the development and advancement of neuropathic pain states [149]. The interplay between oxidative stress and the UPR has emerged as a key driver of pain. Mechanistically, NADPH facilitates ER-resident protein folding by providing the reducing power needed for disulfide bond isomerization, a process easily disrupted by oxidative imbalance [150]. In spinal nerve ligation models, ER stress and oxidative stress engage in a feed-forward loop that regulates pain sensitivity [107]. Central to this is protein disulfide isomerase (PDI), which maintains proteostasis through disulfide bond catalysis but unintentionally contributes to the oxidant load via hydrogen peroxide production. This underscores a nuanced role for PDI in the ER-redox axis. Clinical relevance is further highlighted by findings that PDI can drive hyperalgesia via TRPV1 activation [151].

#### 3.2.4. ER Stress and Apoptosis

The UPR serves as a double-edged sword: moderate signaling supports cell survival, but prolonged activation drives the cell toward apoptosis [152]. In the context of chronic pain, an unresolved UPR fails to restore balance and instead becomes a driver of pathology. This transition contributes to the worsening of chronic pain through persistent inflammatory cycles and neuronal loss. In rats with bone cancer pain, persistent ER stress has been shown to drive neuronal apoptosis in the spinal cord. This cell death is mediated by UPR-induced caspase signaling, which marks a critical transition from cellular stress to permanent damage [104]. XBP1 facilitates the clearance of defective proteins, playing a key role in protecting neurons from the protein-folding stressors found in Alzheimer’s disease [153]. Whether the p-IRE1/XBP1 branch offers the same survival benefits in the context of chronic pain remains an open question that warrants future research.

### 3.3. Cell-Type Specific ER Stress in Chronic Pain

Pain processing involves bidirectional neuronal pathways that convey peripheral noxious inputs via Aδ- and C-fibers to the spinal cord dorsal horn (SCDH) [154]. Nerve trauma disrupts protein homeostasis, inducing ER stress and triggering the UPR in spinal tissues [99,155]. In rodent models of neuropathic pain, robust activation of the primary UPR sensors—IRE1α, PERK, and ATF6—demonstrates comprehensive pathway engagement [107,155]. This response is underscored by neuronal GRP78 induction [99,117], astrocytic eIF2α phosphorylation, and elevated spliced XBP1 expression in the SCDH [99].

Positioned along the spinal cord, the dorsal root ganglia (DRG) serve as critical relays between the peripheral and central nervous systems [156]. The DRG accommodate a heterogeneous cellular population—comprising sensory neurons, satellite glial cells, Schwann cells, and immune cells—dedicated to processing and transmitting peripheral sensory signals. Post-mortem analyses of multiple sclerosis (MS) patients show elevated ER stress markers primarily localized within DRG sensory neurons. Evidence from animal models of painful MS underscores a direct role for ER stress in pain hypersensitivity: expression of ER stress sensors is increased in the DRG, and treatment with the chemical chaperone 4-PBA mitigates pain perception [157]. Furthermore, neuronal ER stress signaling within the DRG is essential for the development of surgery-induced neuropathic pain [158].

## 4. Crosstalk Between ER and Mitochondria in Chronic Pain

Interactions between the ER and mitochondria occur at specialized membrane contact sites termed mitochondria-associated ER membranes (MAMs). As critical sites for ER-mitochondrial crosstalk, structurally complex MAMs regulate diverse physiological and pathological processes, playing a vital role in cellular homeostasis and stress responses. Protein complexes tether the MAM to establish contact sites that drive ER–mitochondria juxtaposition and crosstalk [159]. Architecturally, it relies on the transmembrane anchors ER protein Mmm1 and the outer mitochondrial membrane proteins Mdm10 and Mdm34 to span the inter-organelle space. This conformation is stabilized by the soluble component Mdm12, which binds to the SMP domains of both Mmm1 and Mdm34 [160]. To bridge the ER and outer mitochondrial membranes, the SMP domains of Mdm34, Mdm12, and Mmm1 undergo specific binding. This architecture features a tetrameric hydrophobic channel formed by the head-to-tail dimerization of two Mdm12 and two Mmm1 SMP domains [161]. This unique conformation provides both structural stability and a pathway for signal transduction and the transmembrane transport of phospholipids and Ca^2+^. Multiple protein factors govern this interface: Phosphofurin acidic sorting cluster 2 (PACS2) modulates ER–mitochondrial communication, ER homeostasis, and apoptotic signaling [162], whereas Presenilin 2 (PS2)—operating alongside the outer mitochondrial membrane protein mitofusin 2 (MFN2)—strengthens ER–mitochondrial tethering [163].

### 4.1. Role of MAMs in the ER-Mitochondria Crosstalk

MAMs are characterized by high levels of glucose-6-phosphate phosphatase and lipid synthases, these reversible ER-mitochondrial junctions allow rapid Ca^2+^ transfer into the mitochondria, thereby stimulating ATP production or driving apoptosis [164,165]. Additionally, MAMs function as vital integration centers for glucose and insulin signaling, helping regulate hepatic metabolism and nutrient sensing [166]. MAMs are vital regulatory domains packed with Ca^2+^ channels, lipid synthesis enzymes, and signaling proteins. The breakdown of MAM integrity induces cellular damage via oxidative stress and apoptosis [23,165,167]. This structural dysfunction drives pathology in several conditions; for instance, it contributes to Ca^2+^ dysregulation in Huntington’s disease [166] and disrupts apoptotic pathways in diabetic nephropathy [165]. Structurally, the tethering protein Mfn2 is heavily enriched at these sites, and its loss increases the ER-mitochondrial distance, thereby stunting Ca^2+^ transfer [168,169]. Because MAM structural deterioration is a shared feature in these diseases, repairing ER-mitochondrial crosstalk offers a viable target for therapeutic intervention.

### 4.2. Role of Ca^2+^ in the ER-Mitochondria Crosstalk

The ER and mitochondria function as primary Ca^2+^ reservoirs essential for cellular survival and signaling [21,22]. Proper Ca^2+^ homeostasis within these organellar microenvironments ensures accurate folding of Ca^2+^-dependent proteins such as GRP78 and SERCA [170,171]. While the ER actively keeps baseline cytoplasmic Ca^2+^ levels minimal, controlled mitochondrial Ca^2+^ uptake drives normal signal transduction [172]. However, sustained elevation of cytosolic Ca^2+^ induces mitochondrial permeability transition pore opening, ultimately accelerating cell death [173]. Crosstalk between the ER and mitochondria regulates essential metabolic and survival pathways through controlled Ca^2+^ signaling. ER-to-mitochondrial Ca^2+^ transfer relies heavily on the IP3R-S1R-GRP75-VDAC macromolecular complex. Here, the Sigma-1 receptor (S1R) stabilizes the ER’s IP3 receptor (IP3R) to enable Ca^2+^ efflux, while the chaperone GRP75 bridges this channel to mitochondrial VDAC to facilitate ion uptake [21,159]. Another tethering pair, comprised of ER-localized VAPB and mitochondrial PTPIP51, similarly promotes Ca^2+^ transfer at these contact sites [174]. Transient, localized Ca^2+^ transfer stimulates ATP synthesis and suppresses AMPK-mediated autophagy to maintain energy balance [175,176]. However, prolonged stress shifts this balance: unmitigated ER stress enhances organelle contacts and induces mitochondrial Ca^2+^ overload, driving cells toward apoptosis [167]. On the other hand, inhibiting this pathway—either by blocking ER-mitochondrial Ca^2+^ transfer or knocking out the mitochondrial Ca^2+^ uniporter—severely compromises ATP production, elevates the AMP/ATP ratio, and triggers AMPK-dependent autophagy [177,178,179].

### 4.3. Crosstalk Between ER and Mitochondria in Chronic Pain

Neuronal ER-mitochondrial interactions via the MAM modulate essential nociceptive processes, such as mitochondrial bioenergetics, Ca^2+^ homeostasis, and action potential generation [21,180,181]. Because Ca^2+^ regulates activity-dependent signaling and neuronal excitability, its proper management within nociceptors is vital to suppress aberrant signaling and pain transmission [182]. Thus, targeted Ca^2+^ modulation at the MAM serves as a potential therapeutic avenue for pain control by tuning nociceptor excitability. ER stress drives structural and functional adaptations at the MAM, often increasing contact sites and enhancing inter-organelle Ca^2+^ flux to stimulate mitochondrial energy production [183,184]. Notably, the UPR sensors IRE1 and PERK execute noncanonical roles at this interface [185,186]. While MAM-localized IRE1 interacts with IP3 receptors to sustain mitochondrial bioenergetics [185], PERK regulates ER–mitochondrial proximity and drives apoptosis by conveying ROS signaling and maintaining high CHOP levels [186]. S1Rs are key contributors to neuropathic pain development; indeed, S1R knockout or pharmacological antagonism effectively reduces hypersensitivity in pain models [187,188,189]. In addition to their structural and functional roles at the MAM, S1Rs translocate to the plasma membrane and nucleus to modulate voltage-gated channel kinetics [190]. These distinct cellular mechanisms offer multiple pathways by which S1Rs drive neuropathic pain pathology. The roles of the ER and mitochondria in chronic pain processing are increasingly viewed through the lens of inter-organelle communication. The physical and functional interplay between these organelles is tightly linked to pain signaling; consequently, structural or biochemical disturbances at this interface distort intracellular Ca^2+^ dynamics, serving as a primary driver of nociceptor hyperexcitability [116]. Taken together, in mammalian cells, MAMs regulate Ca^2+^ transfer, lipid metabolism, mitochondrial dynamics, bioenergetics, ROS production, inflammation, and apoptosis. Several mammalian tethering and signaling complexes contribute to MAM function, including IP3R-GRP75-VDAC and Sigma-1 receptor. In neurons and nociceptive pathways, disruption of MAM-dependent Ca^2+^ signaling may promote mitochondrial Ca^2+^ overload, oxidative stress, altered excitability, and neuroinflammation, thereby contributing to pain sensitization.

Human studies provide preliminary but still limited evidence linking organelle stress to chronic pain. Chronic pain is common in patients with mitochondrial disorders [44], while patients with trigeminal neuralgia exhibit increased mitochondrial OXPHOS and oxidative stress in peripheral blood mononuclear cells [50]. In fibromyalgia, reduced mitochondrial bioenergetic health and structural mitochondrial abnormalities have been associated with greater pain or disease severity [191], and elevated circulating ER stress and oxidative stress markers have also been reported [135]. In patients receiving paclitaxel, activation of the ER stress sensor IRE1α in circulating leukocytes was associated with the development and severity of chemotherapy-induced peripheral neuropathy [192]. Nevertheless, these findings are largely based on peripheral biomarkers, small cohorts, and cross-sectional or observational analyses. Direct evidence of altered ER/mitochondrial contact sites in human nociceptive tissues is lacking, and it remains unclear whether these abnormalities are causal, compensatory, or secondary to persistent pain and inflammation.

Uncovering the mitochondrial and ER stress pathways has driven a wide array of targeted therapeutic approaches. Table 1 summarizes these interventions—encompassing small molecules, and natural compounds—organized by their underlying mechanisms, from boosting bioenergetics to tuning mitochondrial dynamics and UPR signaling. Although these preclinical findings offer a strong foundation for clinical translation, mitochondrial dysfunction and abnormal UPR signaling cannot be treated as a uniform condition. Future progress depends on tailored strategies that account for cell-type heterogeneity, disease kinetics, and individual biological variability. Although preclinical findings are promising (Table 1), translating mitochondria- or UPR-targeted analgesics into clinical practice is hindered by significant temporal and biological barriers. First, many effective CIPN strategies—including melatonin and A3AR agonists—depend on prophylactic dosing [65,193,194]. While applicable to scheduled treatments like chemotherapy, prophylactic regimens offer little guidance for treating pre-existing chronic pain. Second, acute rodent models inadequately reflect the long-term pathophysiological trajectory of human pain syndromes. To bridge this translational gap, future studies must focus on late-stage models to test whether targeting mitochondrial pathways can reverse established central sensitization rather than simply masking symptoms.

## 5. Conclusions

This review identifies mitochondrial dysfunction as a primary catalyst in the development of chronic pain. We have mapped the complex interplay of pathological mechanisms that collectively sustain the chronic pain state. A clinical transition from non-specific antioxidant therapies to precision mitochondrial medicine is essential. Targeting discrete molecular switches provides a strategic means of restoring homeostasis without compromising necessary physiological pathways. However, translating these therapies requires solving the challenges of blood-brain barrier permeability and off-target toxicity. Future research must focus on precision delivery tools, like nanotechnology and extracellular vesicles, to achieve effective CNS concentrations. Clinical approaches must be strategically tailored to the stage of the condition. For predictable risks like chemotherapy-induced neuropathy, preventative mitochondrial therapies may reduce the risk of pain development, whereas established chronic pain may require strategies capable of restoring damaged mitochondrial structure and function.

Effective treatments for chronic pain remain limited despite advances in understanding its underlying mechanisms. Preclinical studies now link ER stress to pain signaling, suggesting that therapeutic interventions, such as boosting chaperone activity and preventing UPR-mediated cell death, could offer a promising path forward. However, the precise role of ER stress in chronic pain is still largely unknown. Because most evidence is restricted to animal models, translation to human chronic pain is a major challenge. ER stress contributes to chronic pain pathogenesis by modulating neural circuit remodeling and driving the activation of both astrocytes and microglia. These interactions highlight the role of ER-driven neuroinflammation in maintaining chronic pain states. Future efforts should focus on the cell-type-specific effects of ER stress within pain pathways. Specifically, clarifying how ER dysfunction drives changes in neuronal membrane potential and synaptic excitability will provide deeper insights into the transition to chronic pain.

Beyond primary pain disorders, inflammatory conditions such as multiple sclerosis and rheumatoid arthritis share a common pain-driving architecture rooted in ER stress and mitochondrial dysfunction [195,196]. Disrupted Ca^2+^ signaling may represent unifying pathological mechanisms connecting to these pathways across different diseases. Understanding how mitochondrial dysfunction, ER stress, and ER–mitochondrial crosstalk interact may provide a useful framework for identifying shared molecular drivers of chronic pain and developing more precise therapeutic strategies.

Current findings are not uniform across pain models. Mitochondrial dysfunction is often associated with reduced respiration, membrane potential, and mitophagy [17,49,70,72], whereas increased OXPHOS or mitochondrial activity has been observed in trigeminal neuralgia and inflammatory pain priming [50,54,57]. Notably, mitochondrial activity may decrease during acute inflammation but increase after apparent pain resolution, suggesting a stage-dependent metabolic shift that promotes pain chronification [49,50,73]. Similarly, the UPR or ER stress may initially support cellular adaptation by enhancing ER-mitochondrial coupling and bioenergetics [152,159], but prolonged activation promotes oxidative stress, neuroinflammation, neuronal dysfunction, and apoptosis [99,104,143,148]. These differences likely reflect pain etiology, disease stage, cell type, tissue, and experimental timing. Therefore, mitochondrial and ER stress responses should be viewed as dynamic and context dependent rather than uniformly harmful.

## 6. Future Directions

Despite increasing evidence linking mitochondrial dysfunction and ER stress to chronic pain, several important gaps remain. (1) It is still unclear whether these organelle changes initiate pain chronification, maintain established pain, or act in a stage-dependent manner. (2) The cell-type-specific roles of mitochondrial and ER dysfunction require further clarification. (3) Mitochondrial alterations may represent either bioenergetic failure or maladaptive metabolic remodeling, depending on the pain model and disease stage. (4) The UPR may be protective during acute stress but harmful when chronically activated, and the transition between these states remains poorly defined. (5) ER-mitochondrial communication through MAMs is still underexplored in pain biology. Future studies should identify reliable biomarkers and determine whether targeting mitochondrial dysfunction, ER stress, or MAM signaling can be safely translated into precision therapies for chronic pain. Therefore, mitochondrial changes may reflect either bioenergetic failure or compensatory and maladaptive metabolic activation, whereas the unfolded protein response may be protective during acute stress but harmful when chronically activated. These differences likely depend on the pain model, disease stage, cell type, tissue, and experimental timing. Moreover, most evidence comes from rodent models that re-produce only selected features of human chronic pain and frequently rely on stimulus-evoked withdrawal behaviors rather than spontaneous pain or functional impairment. Clinical translation is further limited by challenges in blood–brain barrier penetration, cell-specific targeting, off-target toxicity, treatment timing, and the absence of validated human biomarkers. Future studies should therefore incorporate clinically relevant behavioral outcomes, both sexes, diverse ages and comorbidities, human tissues, and longitudinal clinical validation. Identifying reliable biomarkers and determining whether mitochondrial dysfunction, ER stress, or MAM signaling can be safely targeted will be essential for developing precision therapies for chronic pain.

## Figures and Tables

**Table 1:** Therapeutic targets related to mitochondrial dysfunction and ER stress in chronic pain.

Targets	Therapeutic Strategy	Pain Model	Limitation
Mitochondrial ROS [27,42]	Reduce mtROS or restore mitochondrial function	neuropathic/inflammatory pain (rodent and human)	specificity, delivery
Mitochondrial bioenergetic dysfunction [26,29,50]	Regulate the mitochondrial dynamics and integrity	neuropathic/inflammatory pain, inflammation-induced orofacial pain (rodent and human)	safety concern, dose determination
NAD^+^/SIRT1 [51]	NAD^+^ precursor supplementation, such as nicotinamide riboside	trigeminal neuropathic pain (mouse)	human validation needed
Mitochondrial quality control [30,74]	Improve mitochondrial transfer, mitophagy, or dynamics	Inflammatory, neuropathic, and orofacial pain models (rodent)	safety concerns
ER stress/UPR signaling [99,104,106]	Chemical chaperones or inhibition of maladaptive UPR pathways	SNL, cancer pain and diabetic neuropathy (rodent)	dose, CNS delivery
ER-mitochondria/MAM signaling [181,182,188]	Modulate Ca^2+^ transfer or Sigma-1 receptor signaling	cancer pain, neuropathic pain (rodent)	maladaptive UPR and cell-type specificity

## Data Availability

No new data were created or analyzed in this study. Data sharing is not applicable to this article.

## Abbreviations

AMPK, AMP-activated protein kinase; ATF4, activating transcription factor 4; ATF6, activating transcription factor 6; BiP, binding immunoglobulin protein; CHOP, C/EBP homologous protein; CIPN, chemotherapy-induced peripheral neuropathy; CoQ10, coenzyme Q10; DRG, dorsal root ganglion; eIF2α, eukaryotic translation initiation factor 2 alpha; ER, endoplasmic reticulum; ERAD, endoplasmic reticulum-associated degradation; GRP75, glucose-regulated protein 75; GRP78, glucose-regulated protein 78; IRE1α, inositol-requiring enzyme 1 alpha; JNK, c-Jun N-terminal kinase; MAM, mitochondria-associated endoplasmic reticulum membrane; MMP, mitochondrial membrane potential; mETC, mitochondrial electron transport chain; mtDNA, mitochondrial DNA; mtROS, mitochondrial reactive oxygen species; mPTP, mitochondrial permeability transition pore; NF-κB, nuclear factor kappa B; NLRP3, NOD-like receptor family pyrin domain-containing protein 3; NRF1, nuclear respiratory factor 1; OXPHOS, oxidative phosphorylation; PDI, protein disulfide isomerase; PERK, protein kinase R-like endoplasmic reticulum kinase; PGC-1α, peroxisome proliferator-activated receptor gamma coactivator 1-alpha; S1R, sigma-1 receptor; SCDH, spinal cord dorsal horn; SIRT1, sirtuin 1; SNL, spinal nerve ligation; TFAM, mitochondrial transcription factor A; TG, trigeminal ganglion; TMJ-OA, temporomandibular joint osteoarthritis; TNF-α, tumor necrosis factor alpha; TRPA1, transient receptor potential ankyrin 1; TRPV1, transient receptor potential vanilloid 1; TUDCA, tauroursodeoxycholic acid; UPR, unfolded protein response; VDAC, voltage-dependent anion channel; XBP1, X-box binding protein 1; XBP1s, spliced X-box binding protein 1.
